# Effect of dance intervention on cardiorespiratory fitness and quality of life of older adults: a systematic review and meta-analysis

**DOI:** 10.3389/fspor.2026.1902108

**Published:** 2026-08-04

**Authors:** Zarifa Kamilova, John Buckley, Bruce Paton, Mike Loosemore

**Affiliations:** 1 Institute of Sport, Exercise and Health, University College London, London, United Kingdom; 2 Sports Medicine Research Laboratory, Azerbaijan Sports Academy, Baku, Azerbaijan; 3 School of Allied Health Professions and Pharmacy, Faculty of Medicine and Health Sciences, Keele University, Newcastle-under-Lyme, Staffordshire, United Kingdom

**Keywords:** cardiorespiratory fitness, dancing, exercise, muscle strength, older adults, physical exercise, quality of life

## Abstract

**Background:**

Physical inactivity is among the dominant risk factors for adverse health outcomes in older adults. It is crucial to investigate effective strategies to encourage older adults to maintain their physical activity. Dancing is recommended as a moderate-intensity activity to enhance cardiorespiratory fitness (CRF) and sustain activity levels. This systematic review and meta-analysis investigates existing trials and synthesizes the effectiveness of a dancing intervention compared with exercising or non-exercising on CRF and quality of life (QoL) in the elderly population.

**Methods:**

The review was conducted according to PERSiST (PRISMA in Exercise, Rehabilitation, Sports Medicine and SporTs Science) guidance. The study protocol was registered with PROSPERO (CRD42024511808). Medline, Embase, Scopus, Science Direct, the Cochrane Library, and Web of Science databases were searched using the following terms: “Danc*/Dancing/Dance therapy/Dance movement therapy; Aging/Aged; Adult/Older adult/Elderly; Chronic disease/Long-term condition; Noncommunicable disease/non-communicable/noncommunicable”. Randomized controlled trials with any style of dance were considered. Outcomes of interest included (1) cardiorespiratory fitness, (2) quality of life, and (3) muscle strength. Date of publication was limited to 2015–2025.

**Results:**

A total of 17 studies involving 1,881 participants (72.85% women) were included. No difference in CRF [standardized mean difference (SMD) −0.89, 95% CI: −1.81–0.03, *p* = 0.0584, *I*^2^ = 87%] and QoL (SMD 0.43, 95% CI −0.15 to 1.00; *p* = 0.14, *I*^2^ = 0%) was found between the dancing and exercising groups. Meta-analysis showed a significant difference in muscle strength favouring dancing compared with the exercising group (SMD 1.64; 95% CI 0.96–2.33; *p* < 0.0001).

**Conclusion:**

This review demonstrated that dance intervention has an effect comparable to that of exercise on improving CRF and QoL in elderly patients. The review also showed that dance is more effective than exercise in improving muscle strength.

**Systematic Review Registration:**

PROSPERO (CRD42024511808), https://www.crd.york.ac.uk/PROSPERO/view/CRD42024511808

## 1 Introduction

Physical inactivity is among the dominant risk factors for adverse health outcomes in the older population (1). European Guidelines on Cardiovascular Prevention (2016) indicate that the recommended minimum activity level is not achieved by the population (2). The World Health Organization (WHO) proposes promoting physical activity as one of the strategies to decrease the burden of chronic diseases worldwide (1).

The American Heart Association (AHA) defines cardiorespiratory fitness (CRF) as the ability of the respiratory and cardiovascular systems to deliver oxygen to skeletal muscles during physical activity (3). Maximal oxygen capacity (VO_2_ max) is considered the gold standard for assessing CRF (4). Low CRF is associated with a higher risk of stroke, heart failure, type 2 diabetes, and cancer (5–8).

Quality of life (QoL) is one of the factors that is highly impacted in the older adult population (9) and defined as individuals’ perception of their position in life in relation to their goals, expectations, standards, and concerns (10). Research shows that chronic heart failure (CHF) patients experience depression and sleeping disorders, resulting in poor QoL (11).

Hand grip strength (HGS), measured with a hand-held dynamometer, is suggested as a new clinical vital sign (12) and is recommended for use as a measurement to identify older adults at risk of poor health status (13). Low HGS is associated with higher rates of hospitalization, mortality, and low QoL (12). HGS is also identified as a physical, behavioural, and psychological factor and is proposed as a health status indicator (14).

According to recent studies, up to 8% of non-communicable diseases (NCDs) in the older population are determined by physical inactivity (15). WHO emphasizes the importance of at least 150–300 min of moderate-intensity physical activity per week for older adults with chronic conditions (16). Therefore, it is crucial to investigate effective strategies to encourage older adults to maintain their physical activity.

AHA recommends dancing as a moderate-intensity activity to enhance CRF and sustain activity levels (17). Dancing is illustrated as beneficial for mental health and QoL in patients with cancer and Parkinson’s disease (18). Dancing is described as a holistic intervention that supports emotional well-being and social engagement among elderly people (19, 20).

To our knowledge, the last systematic review on the effect of dancing on cardiovascular risk of aging population was conducted in 2016 (21). The results demonstrated that dancing was as effective as other forms of exercise and was considered an alternative to exercise interventions for improving CRF. A meta-analysis assessing of QoL in older adults was conducted in 2021, but only participants with mild cognitive impairment were included (22). The authors concluded that dance has a significant effect on QoL.

This systematic review and meta-analysis aims to synthesize the effectiveness of a dancing intervention compared with exercising or non-exercising on CRF and QoL in older adults. It also summarizes the evidence on the intensity, frequency, and duration of the dancing intervention.

## 2 Methods

### 2.1 Protocol and registration

This review followed “PRISMA-S: an extension to the PRISMA Statement for reporting Literature Searches in Systematic Reviews” (23) and “The PRISMA 2020 statement: an updated guideline for reporting systematic reviews” (24). The PERSiST (PRISMA in Exercise, Rehabilitation, Sports Medicine and SporTs Science) guidance was also considered (25). The study protocol was registered with PROSPERO in February 2024 (CRD42024511808). Data extraction was completed in December 2025.

### 2.2 Eligibility criteria

The PICOS (Population, Intervention, Comparison, Outcome, Study design) framework was used to define the inclusion and exclusion criteria. Randomized controlled trials (RCTs) involving any style of structured dance delivered individually or in groups on a weekly basis, with reported session design and duration, were eligible for inclusion. Study participants were defined as subjects exposed to the dancing intervention. Controls included participants who were involved in traditional exercises, walking activities, or who were not exercising (waiting list). Outcomes of interest included (1) cardiorespiratory fitness, (2) quality of life, and (3) muscle strength. No language restrictions were imposed to ensure broader coverage of the existing literature. Studies published between 2015 and 2024 were included, as the most recent systematic review on this topic covered literature up to 2015 (21). The final search in December 2025 additionally specified the publication year range as January 2024–December 2025. Exclusion criteria were identified as follows: (1) age below 55, (2) dance as part of any other intervention, (3) no outcome of interest, and (4) studies other than RCTs.

### 2.3 Search strategy

RCTs reporting the effect of a dance intervention on CRF, QoL, and muscle strength of older adults were searched. A specialist librarian and scientific advisor validated the search strategy. Preliminary searches in PubMed using the terms “dance,” “older adults,” and “chronic disease” helped identify relevant medical subject heading (MeSH) terms. These terms, “Danc*/Dancing/Dance therapy/Dance movement therapy; Aging/Aged; Adult/Older adult/Elderly; Chronic disease/Long-term condition; Non-communicable disease/non-communicable/noncommunicable,” were then used to search Medline, Embase, Scopus, Science Direct, the Cochrane Library, and Web of Science databases. The reference lists of included studies were manually screened to identify additional articles. Two reviewers independently screened all papers against predefined eligibility criteria. Disagreements resolved through discussion. Where consensus could not be reached, a third reviewer was involved. One study author was contacted to obtain a full text (26). No grey literature search was conducted. The final search, using the same strategy and databases, was performed in December 2025. The complete search strategy is provided in Supplementary Material 1.

### 2.4 Risk of bias assessment of RCTs’

The Cochrane Risk of Bias (RoB) 2 tool for assessment risk of bias in randomised controlled trials (RoB 2.0 tool) was used to evaluate the quality of RCTs (27). Assessment was performed based on the “Intention to treat” approach for all five domains: (1) randomization process, (2) deviations from intended interventions, (3) missing outcome data, (4) outcome measurement, and (5) selection of the reported result. An overall RoB judgement was made for each outcome as either “low risk,” “some concerns,” or “high risk” of bias. The RoB assessment was carried out independently by one assessor and verified by a second one. Any discrepancies in the quality appraisal were discussed between the authors until a consensus was reached.

### 2.5 Data extraction and synthesis

All records retrieved from the databases were imported into EndNote (version 21.2). Duplicates were removed using the Deduplicator tool from the Systematic Review Accelerator (https://sr-accelerator.com/#/deduplicator). Titles and abstracts were primarily screened, and full texts were assessed when abstracts did not provide sufficient information. Data were then extracted from all eligible studies, including title, authors, publication year, study design, sample size, mean age, and disease. Data on the outcomes of interest (CRF, QoL, and muscular strength) and the measurement tools were recorded. Two authors independently collected the data from papers.

#### 2.5.1 Statistical analysis plan

Effect sizes in RCTs were calculated as standardized mean differences. A random-effect model was used to estimate the average distribution of treatment effects and to calculate 95% confidence and prediction intervals. Subgroup analyses were not conducted because only a limited number of papers were identified. Sensitivity analyses were not conducted because we did not identify any studies with low risk of bias. Heterogeneity was evaluated using the tau-squared and *I*^2^ statistics. *I*^2^ values above 75% were considered high heterogeneity. All analyses were conducted and displayed in R, version 4.3.1 (R Core Team, 2023).

### 2.6 Equity, diversity, and inclusion

Trials conducted across diverse geographic regions were included in the review. No language restrictions were applied. Most of the participants were women (72.85%) and had various NCDs. The authors comprised junior and senior investigators from multiple countries and varied backgrounds, including one female researcher. This multidisciplinary and international composition may enhance the generalizability of the findings and reduce potential bias.

## 3 Results

### 3.1 Characteristics of the included studies and participants

**Figure 1:**
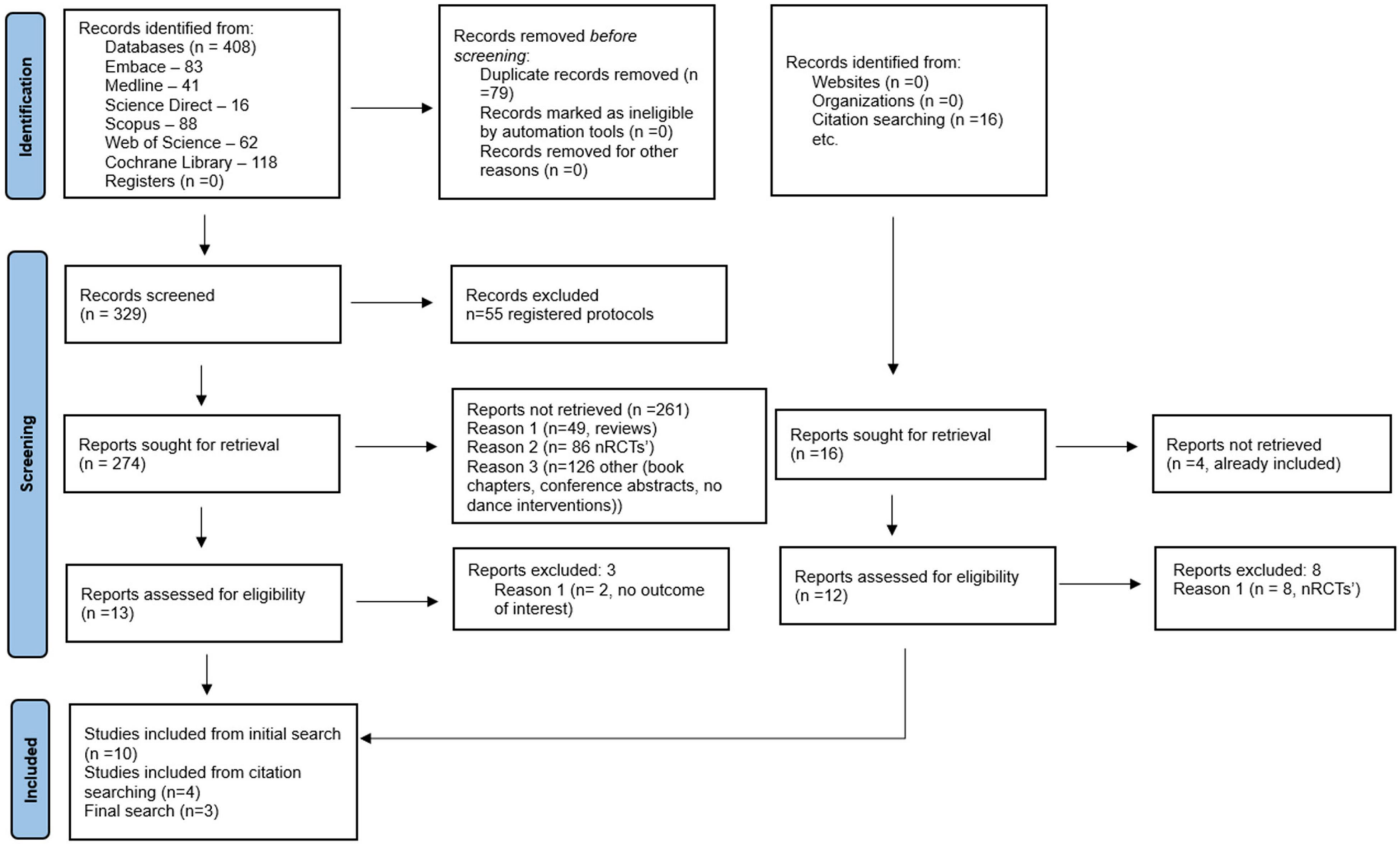
PRISMA flow diagram demonstrating searching for studies. From: Page et al. *BMJ*. (2021) 10(1):89. doi: 10.1186/s13643-021-01626-4. (24).

A total of 408 papers were initially identified from 7 databases, of which 79 were removed due to duplication. The titles and abstracts of the remaining 329 studies were screened manually. After excluding non-eligible studies (registered protocols, non-RCTs, and reviews), 10 records remained by the end of the screening process. Reasons for exclusion at the full-text screening stage were recorded according to the PRISMA 2020 recommendation. The reasons for exclusion included reviews, non-randomized controlled trials, and book chapters. Manual searching of the reference lists identified an additional 16 studies, of which four were included in the review list (28–31). Three studies were added as a result of the final search (32–34). The PRISMA flow diagram of the study selection process is provided in Figure 1. Table 1 presents the characteristics of the studies.

**Table 1:** Characteristics of the studies included in the review.

*N*	Author, year	Total no. of participants	Females (%)	No. of participants in dancing group	Age	Condition (disease)	Outcome	Measurement tool	Duration and frequency of the intervention
1	Broscheid K., 2020 (35)	24	75	14	70.8 + 10.6	Lumbar Spinal Stenosis, decompression laminectomy	QoL	ODI	60 min, 2x week, 6 weeks
2	Burzynska A., 2017 (28)	174	69	49	65.4 (4.46)	Low active older adults	CRF	Max Exercise Testing (VO2 peak)	60 min, 3x week, 6 months
3	Cruz-Ferreira A., 2015 (36)	78	100	32	71.1 + 3.9	Older females	CRF, Muscle Strength	6MWT, 30s CST	50 min, 3x week, 24 weeks
4	Eggenberger P., 2015 (37)	89	16	30	Over 70	0, 1, or 2 within the Swiss classification system for health care requirements	CRF, Muscle Strength	6MWT, 5 Chair Rises Test	60 min, 2x week, 26 weeks
5	Esmail A., 2020 (38)	62	75.2	23	67.48 ± 5.37	Inactive elderly	CRF, QoL	Rockport One-Mile test (MAP), SF-12	60 min, 3x week, 12 weeks
6	Kaholokula, 2017 (30)	55	86	27	55 (10)	Hypertension (SBP >140 or >130 if have co-morbid diabetes)	CRF, QoL	6MWT, SF-12	60 min, 2x week, 12 weeks
7	Machacova K., 2017 (31)	189	90	92	82.95 ± 8.56	Older adults	CRF, Muscle Strength	2 min step-test, Arm Curle Test, Chair Stand Test	60 min, 1x week, 12 weeks
8	Merom D., 2016 (39)	552	85	279	69.5 ± 6.4	Older adults	QoL	SF-12	60 min, 2x week, over 12 months
9	Merom D., 2016 (40)	115	76.7	60	69.5 ± 6.4	Older adults	CRF	6MWT	60 min, 2x week, 8 months
10	Muller P., 2017 (41)	52	45	26	68.25 (3.91)	Elderly individuals	CRF	PWC 130 test	90 min, 2x week, 6 months
11	Rehfeld K., 2018 (29)	52	52	26	63–80	Older adults	CRF	PWC 130 test	90 min, 2x week, 6 months
12	Sanprakhon P., 2025 (34)	108	82–88	56	69.82 ± 6.18	Subjective cognitive decline	QoL	WHOQOL-BREF	90 min, 2x week, 7 weeks
13	Serrano-Guzman M., 2016 (32)	52	100	27	69.7 ± 3.85	Sedentary postmenopausal women	CRF, QoL	IFIS, SF-12	50 min, 3x week, 2 months
14	Tung H., 2024 (33)	60	78	30	68.3 ± 5.1	Older adults	QoL	EQ-5D	30 min, 2x week, 6 months
15	Vrinceanu, T., 2019 (42)	40	72.2	12	67.45	Older adults	CRF, QoL	MAP, SF-12	60 min, 3x week, 3 months
16	Vordos, Z., 2017 (43)	40	–	20	73.2 ± 4.7	Chronic Heart Failure	CRF, Muscle Strength	6MWT, leg-chest dynamometer	45–60 min, 3x week, 12 weeks
17	Woloszyn N., 2023 (44)	169	58	57	74.4	Functionally limited older adults	Muscle Strength	Hand Grip Strength, Arm Curle test	30 min, 2x week, 12 weeks

SBP, systolic blood pressure; CST, chair stand test; CRF, cardiorespiratory fitness; QoL, quality of life; ODI, Oswestry Disability Index; 6MWT, 6-min walk test; 30-s chair stand test; MAP, maximal aerobic power; Short-Form 12; PWC, physical working capacity; WHOQOL-BREF, World Health Organization Quality of Life Brief Scale; IFIS, International Fitness Scale; EuroQol 5-Dimensions.

The total number of participants from the 17 studies is 1,881, with 72.85% women. The sample size ranged from 24 to 522 older adults, with a mean of 111 participants. The mean age of participants was 70.8(+/−6.09) years. Eight studies participants involved older adults without serious health conditions (1,095, 58.2%). Four studies involved participants with different non-communicable health conditions such as CHF, lumbar spinal stenosis, hypertension, and cognitive decline (227, 12%). Five studies did not report any health condition (559, 29.8%). Overall, 860 (45.7%)participants took part in dancing interventions, while 54.3% of participants served as exercise and non-active controls. Table 2 describes the duration and frequency of dancing intervention in the included studies.

**Table 2:** Duration and frequency of dancing intervention in studies included in the review.

Duration (weeks)	No. of studies, *n* (%)	Frequency (per week)	No. of studies, *n* (%)	Total duration (h)	No. of studies, *n* (%)
6–48	6 (35.3)	2	10 (58.8)	>24	8 (47.1)
12	6 (35.3)	3	6 (35.3)	25–50	2 (11.8)
24	5 (29.4)	1	1 (5.9)	51–65	3 (17.6)
				66–80	3 (17.6)
				<80	1 (5.9)

### 3.2 Risk of bias assessment

Two reviewers independently assessed the risk of bias of the included trials using the Cochrane Risk of Bias (RoB 2) tool (45). Agreement between the assessors on methodological quality was 86%. Discrepancies were resolved through discussion. The trials were judged to be at high risk of bias if at least one domain was rated as high risk, at some risk if at least one domain raised some concerns, but none were high risk. The studies were rated as low risk if all domains were evaluated as low risk.

**Figure 2:**
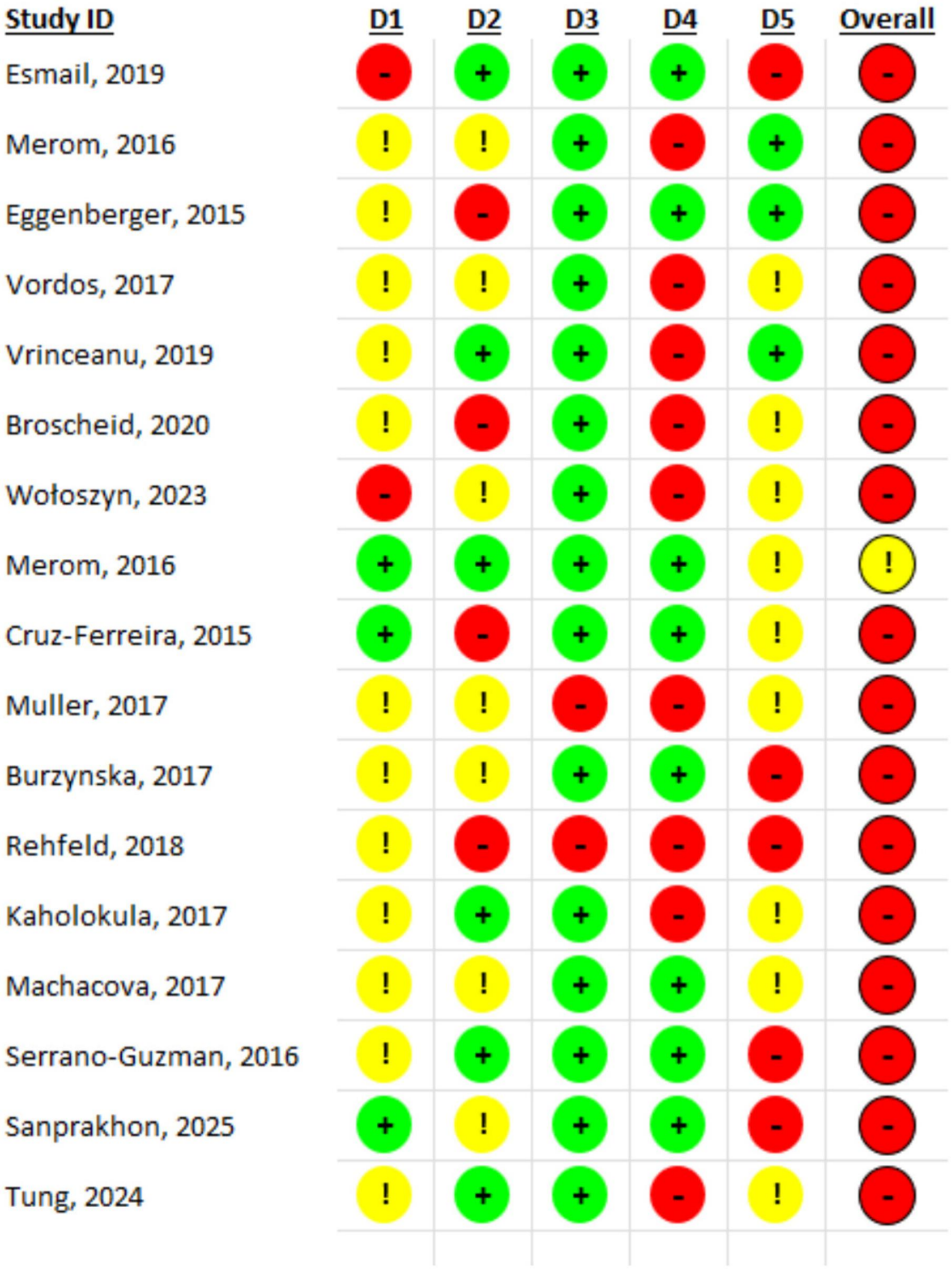
Risk-of-bias assessment of included papers. 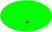, low risk; 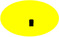, some concerns; 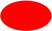, high risk; D1, Randomization process; D2, deviations from the intended interventions; D3, missing outcome data; D4, measurement of the outcome; D5, selection of the reported results.

Sixteen studies (94%) were at high risk of bias. One study (6%) was at some risk of bias. No trials were at low risk of bias. Most of the biases arose in the domains of measurement of the outcome (D4, 52.9%), selection of the reported results (D5, 29.4%), and deviation from the intended intervention (D2, 23.5%). Two studies were rated as having a high risk of bias in Domain 3 (missing outcome data) (29, 41) because of more than 20% missing outcome data (37% and 25% dropout rate, respectively), exceeding the threshold considered as “large” (over 20%) (45). The evidence that missingness was unrelated to the true outcome was insufficient. Two studies (38, 44) did not provide detailed information regarding allocation concealment within the randomization process domain (D1) and were rated as high risk in Domain 1. No studies were excluded based on the risk-of-bias assessment, as this would decrease the number of eligible studies for meta-analysis. Risk-of-bias assessment results are shown in Figure 2 and Supplementary Material 2.

### 3.3 Effect size of dance intervention

The review examined the effect size of dance intervention on CRF, QoL, and muscle strength. A random-effects model was used to conduct a meta-analysis. Subgroup analysis and publication bias were not conducted due to an insufficient number of studies. Sensitivity analyses excluding high-risk studies were not conducted because a limited number of eligible RCTs were identified (45). Based on that, findings were interpreted with appropriate caution. We analysed heterogeneity among studies quantitatively using the *I*^2^ test. Two meta-analyses were conducted to compare the effects of dance intervention versus non-exercising controls and dance intervention versus walking controls on CRF. Another two meta-analyses compared the effect of dance intervention versus exercising and non-exercising controls on QoL. Additional meta-analysis was conducted to compare the effect of dancing on muscle strength.

#### 3.3.1 Cardiorespiratory fitness

Twelve studies assessed CRF. Five studies used the same measurement tool (6-min walk test, 6MWT). Three studies were excluded from the meta-analysis for (1) using a subjective tool (32), (2) assessing CRF only on the pre-intervention stage (28), and (3) not reporting the results in the paper (29). The author of the latter also did not respond to the emails. The percentage change in the standardized mean difference (SMD) and standard error was calculated to pool nine studies for the meta-analysis. Three studies used a two-arm design comparing dancing with exercise controls (37, 40, 41). Four studies compared a dance intervention with non-exercising controls (30, 31, 36, 43). Two trials were designed as a three-armed intervention using dance, exercise, and non-exercising groups (38, 42). Three authors were contacted by email to seek clarification on issues of reporting and missing data (29, 38, 42). One author did not respond (29).

**Figure 3:**
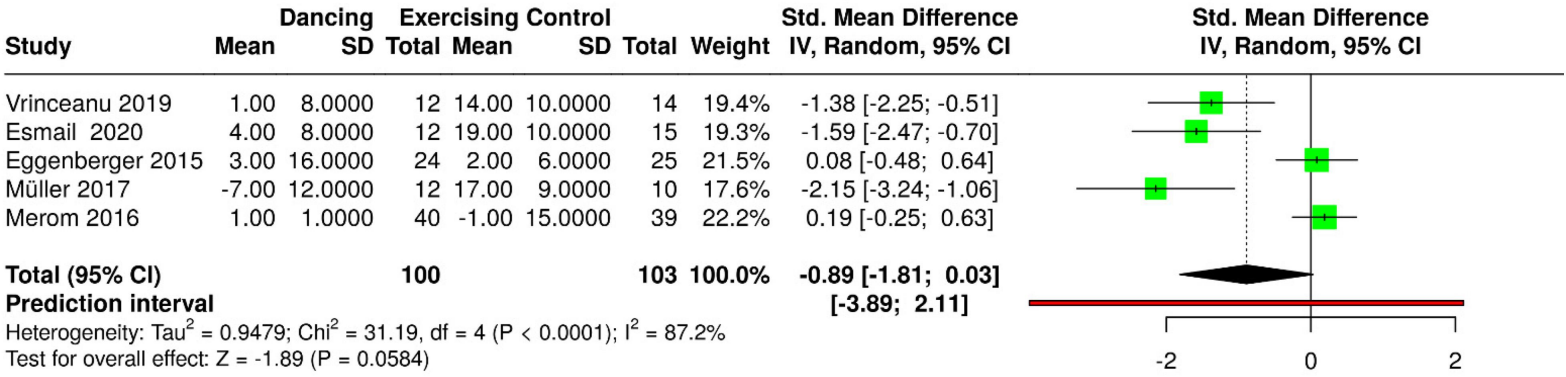
Change in cardiorespiratory fitness in the dance and exercise groups.

**Figure 4:**
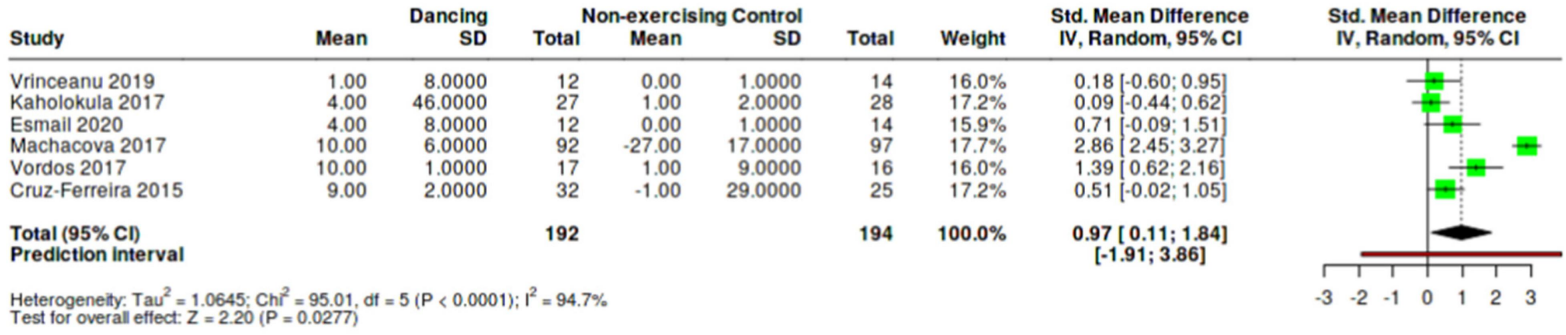
Change in cardiorespiratory fitness in the dance and non-exercising groups.

A meta-analysis of five studies on dance and exercise cohorts (37, 38, 40–42), including 203 participants, was performed using a random-effects model. No significant difference in CRF was found between the groups (SMD −0.89, 95% CI: −1.81–0.03, *p* = 0.0584, *I*^2^ = 87%) (Figure 3). Six studies (30, 31, 36, 38, 42, 43) comparing dance and non-exercising controls were analysed, with a total of 386 participants. Based on the analysis using a random-effects model, a statistically significant difference was found between the two cohorts (SMD 0.97, CI: 011–1.84, *p* = 0.0277, *I*^2^ = 94.7%) (Figure 4).

#### 3.3.2 Muscle strength

**Figure 5:**
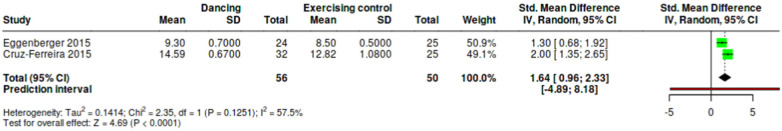
Change in muscle strength in the dance and exercise groups.

**Figure 6:**
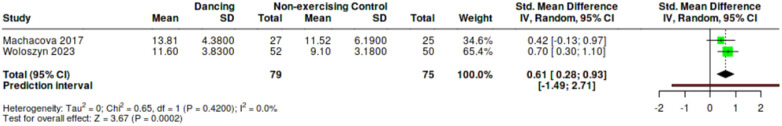
Change in muscle strength in the dance and non-exercising groups.

Two studies comparing dance and exercise groups evaluated muscle strength using the chair stand test. The total number of participants was 106. A meta-analysis revealed a significant difference favouring dancing (SMD 1.64; 95% CI 0.96–2.33; *p* < 0.0001), with moderate heterogeneity (*I*^2^ = 57.5%, *p* = 0.1251) (Figure 5). We also compared muscle strength between dance and non-exercising groups. Two studies using the arm curl test were analysed, with 79 participants in the dance cohort and 75 in the non-exercising control cohort (31, 44). There was a statistical difference between the two cohorts, favouring the dancing intervention (SMD 0.61, 95% CI 0.28–0.93, *p* = 0.0002). No heterogeneity was detected (*p* = 0.4200) (Figure 6).

#### 3.3.3 Quality of life

Nine studies, involving a total of 1,001 participants, assessed QoL. Five studies (*n* = 731) using the Short Form 12 (SF-12) scale were pooled for the meta-analysis (30, 32, 38, 39, 42). As the physical and mental component domain scores were reported separately across included studies, independent meta-analyses were conducted for each domain. Comparisons were performed between the dance intervention and both the exercise and non-exercising groups.

No statistically significant differences in QoL were found for SF-12 physical or mental domains. For the physical component, the pooled SMD was −0.05 (95% CI −0.20–0.10; *p* = 0.52, *I*^2^ = 0%) when comparing dance with non-exercising controls and 0.43 (95% CI −0.15–1.00; *p* = 0.14, *I*^2^ = 0%) when comparing dance with the exercise control cohort. Similar non-significant results were observed for the mental component: SMD was 0.09 (95% CI −0.18–0.35; *p* = 0.52, *I*^2^ = 24.2%) for dance vs. non-exercising group and 0.18 (95% CI −0.36–0.72; *p* = 0.52, *I*^2^ = 0%) for dance vs. exercise group (Supplementary Material 3).

## 4 Discussion

The current systematic review and meta-analysis aimed to investigate the effectiveness of a dancing intervention on CRF and QoL of older adults. Our meta-analysis showed that the dancing intervention has the same effectiveness on CRF and QoL as traditional exercise.

The review primarily aimed to investigate the effectiveness of dance on CRF and QoL in patients with chronic NCD. The initial search identified an insufficient number of published studies analysing the relevant topic since 2015. Only one RCT analysed the effect of a dance intervention on VO_2_ max in CHF patients (43). We therefore decided to broaden this systematic review and meta-analysis to investigate the effects of dance interventions on CRF and QoL in older adults, with and without various NCDs.

This review is not the first to analyse the effectiveness of dance interventions on cardiorespiratory outcomes. Neto et al., in a systematic review and meta-analysis, investigated the effect of dance therapy in patients with CHF (46). Neto’s review includes two RCTs with 62 participants. Rodrigues-Krause conducted a systematic review and meta-analysis summarising RCTs and non-randomized controlled studies investigating the effect of dance on cardiovascular risk factors in older adults (21). The total number of participants included in seven papers (six RCTs and one nRCT) was 377. In our review, we summarized 12 RCTs with a cardiorespiratory outcome and a total of 998 study participants, of which 424 (42.4%) were randomized to dancing interventions. Nine of these papers, comprising 322 participants involved in dancing, were pooled for the meta-analysis.

### 4.1 Cardiorespiratory fitness

Maximal oxygen consumption (VO_2_ max, mL/min/kg) is recognized as the gold standard measure of CRF (4). Among the 12 studies assessing CRF in this review, only two used VO_2_ max as an indicator of CRF (28, 43). Only one study conducted pre- and post-intervention VO_2_ max assessments (43). In contrast, Burzynska et al. evaluated VO_2_ max only at baseline in a low-active older adult population, without post-intervention follow-up (28). Five trials assessed CRF using the 6MWT (30, 36, 37, 40, 43). Vordos et al. reported the largest improvement in walking distance—approximately 10%—following a 12-week Greek dance intervention in patients with CHF (43). The author links this improvement to cardiorespiratory adaptations resulting from the intervention (43). In contrast, Merom et al. found no significant improvement in 6MWT performance after a 32-week dance program in adults aged 60 or older (40). The authors explained that their participants were highly active at baseline, resulting in smaller improvements and lowering the effect size (40). This lack of effect may be explained by high activity levels of participants at baseline and by methodological inconsistencies: the program was delivered across five studios, by five instructors, and involved multiple dance styles (Rock and Roll, Foxtrot, Waltz, and Latin). Because intensity was not monitored objectively, standardization and comparability across sessions were uncertain, reducing the effect size (40). Consistent with the findings of Neto et al., who concluded in their systematic review and meta-analysis that dance therapy is effective compared with non-active controls (46), our analysis similarly found that dance significantly outperforms non-exercise comparators.

Chen et al. used the 6MWT to measure aerobic capacity and demonstrated significant improvements in CHF patients following a 3-month exercise-based cardiac rehabilitation program compared with non-exercising controls (18.2 ± 4.1 vs. 20.9 ± 6.6 mL/min/kg, *p* = 0.02) (47). The same study also reported significant increases in walking distance (421 ± 90 vs. 462 ± 74 m, *p* = 0.03). Our review also demonstrated the effectiveness of the dancing intervention compared with the non-exercising control. These findings suggest that dancing may be an alternative to exercise for improving CRF.

Substantial heterogeneity was observed among studies assessing CRF, with values of 87.2% and 94.7% for dancing/exercising and dancing/non-exercising comparisons, respectively. To better understand this variability, we examined characteristics of the dance interventions. The largest improvements were observed in Vordos et al. and Machacova et al. (31, 43). Vordos et al. reported a 10% increase in 6MWT distance among CHF patients aged 70 or older, with New York Heart Association (NYHA) Classes I and II (43). As outlined in the European Society of Cardiology guidelines for the diagnosis and treatment of acute and CHF (2021), these patients have no or only slight limitations in physical activity, making them more likely to tolerate and respond to moderate-intensity dance intervention (48).

Vordos also referenced Kaltsatou et al., who observed over 20% improvement in CRF after a longer 32-week (96 h) Greek dance rehabilitation program (49). This suggests that program duration and total exercise hours may play an important role in improving CRF.

Machacova et al. reported similar improvements (10%) despite offering only weekly sessions over 3 months (12 total hours) to participants with a higher mean age (82.95 ± 8.56) (31). These results may be explained by increased intensity of dancing activities for each patient, according to their abilities, although intensity was judged subjectively by the dance instructor rather than measured objectively. Cruz-Ferreira et al. also observed a 9.4% improvement in aerobic capacity (36). Despite similarities in participant age compared with Vordos, differences in intervention duration [a 24-week (60-h) program in Cruz-Ferreira vs. a 12-week (39-h) program in Vordos] may explain variability in outcomes.

Differences in inclusion criteria may further contribute to heterogeneity. Vordos focused on older adults with diagnosed CHF, whereas Cruz-Ferreira included generally healthy older adults, and Machacova did not report participants’ health conditions in detail. In addition, the use of different CRF assessment tools (the 2-min step test vs. the 6MWT) likely introduced methodological variability.

### 4.2 Quality of life

Dance intervention may influence QoL through several mechanisms. Dance is primarily delivered in group settings (40). Group-based physical activities (50) are known to enhance psychological well-being and promote social interaction (51). Dancing can also improve emotional and physical capacities (52). Despite these scientifically proven benefits, our review found no significant effect of the dance intervention on QoL compared with non-exercising controls across the five included trials.

The largest study in our analysis was conducted by Merom et al., which contributed 275 of 335 participants to the meta-analysis (40). Although this study showed the largest effect size (80.5%), the difference between groups was not statistically significant. Several factors may explain this. First, the dance group participants were, on average, older and had lower baseline cognitive scores than the control group, which may have influenced their QoL outcomes. Second, the intervention lasted over 48 weeks, and participant' QoL may have declined naturally over this extended period due to aging. In addition, although the program was prescribed as standardized, it was delivered by eight different instructors, which could have introduced variability in intervention quality (39).

Our findings align with the results of Wu et al., who reported no significant effect of dance interventions on QoL when assessed with the Short Form 36 (SF-36) questionnaire (22). Wu observed a moderate, statistically significant effect when QoL was measured using the shorter SF-12 scale. This discrepancy may arise from the questionnaire length: the SF-12 is shorter and easier to complete, resulting in more reliable responses in older adults. In our review, after pooling only studies that used the SF-12, we still found no significant difference in QoL between the dance/exercise or dance/non-exercising groups.

By contrast, Neto et al. found that dance therapy significantly improved QoL in heart failure patients compared with non-exercising controls, although no significant difference was observed between dance and traditional exercise (46). While Neto concluded that dance is as effective as exercise and may serve as an alternative, the reliability of these findings is methodologically limited. Only two trials were included in the review, and they used different instruments (SF-12 and the Minnesota Living with Heart Failure Questionnaire). In contrast, our review included five studies, all using the same QoL measurement tool (SF-12), which provides more evidence-based results.

### 4.3 Muscle strength

Our meta-analysis showed that dancing was more effective than exercising and non-exercising in improving muscle strength. The evidence suggests exercise improves muscle strength in older adults (53). HGS has been shown to be a validated predictor of chronic conditions (54). In our review, only one study used HGS to assess strength (44). Two studies reported significant improvement in muscle strength following a 12-week dance intervention (31, 44). Our meta-analysis found that a 3-month dance intervention significantly increases muscle strength. Compared with our results, Eggenberger et al. reported a reduction in muscle strength after 3 months in the dance and exercise groups (37). They observed a greater decline in the exercise group, attributing it to low-training intensity and larger group sizes. We suggest a smaller decline in muscle strength in dance group participants due to a greater protective effect of dance against age-related strength loss. The significant heterogeneity, potentially caused by variation in strength assessment tools, limits the certainty of these findings.

### 4.4 Limitations

This review identified several limitations. First, outcomes were measured using various tools and scales, making it difficult to synthesize the results for meta-analysis. There were insufficient numbers of studies using cardiopulmonary exercise testing (CPET) to allow strong conclusions about the effect of dance on CRF. Alternative measures, such as the 6MWT, used in the included trials are not interchangeable with CPET. Significant heterogeneity was observed across studies in the included participants, dance type, programme design, duration, and frequency, preventing clear recommendations on optimal training protocols. This fact limited the review process; therefore, we deviated from the registered protocol to achieve sufficient study numbers. As an amendment to the registered protocol, healthy older adults were included because the number of studies involving NCD populations was insufficient for the meta-analysis.

In addition, none of the included trials reported the intensity of the dancing sessions or measured heart rate or oxygen consumption, limiting the interpretation of aerobic demands. The overall quality of evidence was weakened by low-quality RCTs, as assessed by the Cochrane Risk of Bias assessment. Finally, reporting on safety measures and adverse events was limited, which is essential, suggesting that NCD populations are considered high-risk.

### 4.5 Practical recommendations

Future research should prioritize RCTs that use standardized outcome measures. CPET is recommended for assessing CRF. Validated tools such as HGS for muscle strength should also be considered. Study characteristics, such as frequency and duration, should be reported. The intensity of the dancing intervention should be incorporated to better define the exercise dose. Greater methodological consistency is also required to reduce heterogeneity.

In clinical practice, dancing may be considered as a feasible and engaging alternative to traditional exercise for older adults to maintain physical function and promote adherence to physical activity. Programmes of moderate duration (approximately 12–24 weeks), delivered 2–3 times per week, may be appropriate based on current evidence. Clinicians are recommended to ensure appropriate supervision, monitor the intensity of dancing, and implement safety measures, especially in high-risk populations. Given the high heterogeneity in reported outcomes, dance intervention can be integrated as an additional approach rather than a replacement for traditional exercise programmes until more robust scientific evidence becomes available.

## 5 Conclusion

This review demonstrated that dance intervention has an effect comparable to that of exercise on improving cardio-respiratory fitness and quality of life in the elderly population. A meta-analysis found that dance is more effective than exercise in improving muscle strength. The meta-analysis also demonstrated that dance intervention significantly improves CRF and muscle strength compared with non-exercising controls.

## Data Availability

The original contributions presented in the study are included in the article/Supplementary Material, further inquiries can be directed to the corresponding author.
