# Supplementary material for: Effect of dance intervention on cardiorespiratory fitness and quality of life of older adults: a systematic review and meta-analysis
Source: Front Sports Act Living. 2026 Aug 4;8:1902108. doi: 10.3389/fspor.2026.1902108 (PMC13481374; doi:10.3389/fspor.2026.1902108)
Supplement: Supplementary file 1 [file Datasheet1.docx]

Quality of Life (Dancing/Non-Exercise) SF-12 Physical Domain

**Conclusion:**

- All together 5 studies were analyzed with a total of 353 subjects in the Dancing cohort and 330 subjects in the Non-Exercising Control cohort.
- Based on the analysis performed using random effects model with Inverse variance method to compare the standardized mean difference (SMD), there is no statistical difference between the two cohorts, the summarized standardized mean difference (SMD) is -0.05 with a 95% confidence interval of -0.2 - 0.1.
- The test for overall effect does not show a significant effect.
- No significant heterogeneity was observed, suggesting that the effect sizes across studies remained uniform in both scale and direction.

Quality of Life (Dancing/Non-Exercise) SF-12 Mental Domain

**Conclusion:**

- All together 5 studies were analyzed with a total of 353 subjects in the Dancing cohort and 330 subjects in the Non-Exercising Control cohort.
- Based on the analysis performed using random effects model with Inverse variance method to compare the standardized mean difference (SMD), there is no statistical difference between the two cohorts, the summarized standardized mean difference (SMD) is 0.09 with a 95% confidence interval of -0.18 - 0.35.
- The test for overall effect does not show a significant effect.
- Notable variability was not detected, signaling that the effect sizes across cohorts remained uniform in both scale and direction.

Quality of Life (Dancing/Exercise) SF-12 Physical Domain

**Conclusion:**

- All together 2 studies were analyzed with a total of 24 subjects in the Dancing cohort and 24 subjects in the Exercising Control cohort.
- Based on the analysis performed using random effects model with Inverse variance method to compare the standardized mean difference (SMD), there is no statistical difference between the two cohorts, the summarized standardized mean difference (SMD) is 0.43 with a 95% confidence interval of -0.15 - 1.
- The test for overall effect does not show a significant effect.
- We did not detect notable variability, signaling that the effect sizes across cohorts were uniform in both magnitude and direction.

Quality of Life (Dancing/Exercise) SF-12 Mental Domain

**Conclusion:**

- All together 2 studies were analyzed with a total of 24 subjects in the Dancing cohort and 29 subjects in the Exercising Control cohort.
- Based on the analysis performed using random effects model with Inverse variance method to compare the standardized mean difference (SMD), there is no statistical difference between the two cohorts, the summarized standardized mean difference (SMD) is 0.18 with a 95% confidence interval of -0.36 - 0.72.
- The test for overall effect does not show a significant effect.
- Significant heterogeneity was not observed, signaling that the effect sizes across cohorts were uniform in both magnitude and direction.
